# Comparison of the progressive resolution optimizer and photon optimizer in VMAT optimization for stereotactic treatments

**DOI:** 10.1002/acm2.12355

**Published:** 2018-05-20

**Authors:** Han Liu, Benjamin Sintay, Keith Pearman, Qingyang Shang, Lane Hayes, Jacqueline Maurer, Caroline Vanderstraeten, David Wiant

**Affiliations:** ^1^ Department of Radiation Oncology Cone Health Cancer Center Greensboro NC USA

**Keywords:** brain SRS, lung SBRT, optimization, VMAT

## Abstract

The photon optimization (PO) algorithm was recently released by Varian Medical Systems to improve volumetric modulated arc therapy (VMAT) optimization within Eclipse (Version 13.5). The purpose of this study is to compare the PO algorithm with its predecessor, progressive resolution optimizer (PRO) for lung SBRT and brain SRS treatments. A total of 30 patients were selected retrospectively. Previously, all the plans were generated with the PRO algorithm within Eclipse Version 13.6. In the new version of PO algorithm (Version 15), dynamic conformal arcs (DCA) were first conformed to the target, then VMAT inverse planning was performed to achieve the desired dose distributions. PTV coverages were forced to be identical for the same patient for a fair comparison. SBRT plan quality was assessed based on selected dose–volume parameters, including the conformity index, *V*_20_ for lung, *V*_30 Gy_ for chest wall, and *D*_0.035 cc_ for other critical organs. SRS plan quality was evaluated based on the conformity index and normal tissue volumes encompassed by the 12 and 6 Gy isodose lines (*V*_12_ and *V*_6_). The modulation complexity score (MCS) was used to compare plan complexity of two algorithms. No statistically significant differences between the PRO and PO algorithms were found for any of the dosimetric parameters studied, which indicates both algorithms produce comparable plan quality. Significant improvements in the gamma passing rate (increased from 97.0% to 99.2% for SBRT and 96.1% to 98.4% for SRS), MCS (average increase of 0.15 for SBRT and 0.10 for SRS), and delivery efficiency (MU reduction of 29.8% for SBRT and 28.3% for SRS) were found for the PO algorithm. MCS showed a strong correlation with the gamma passing rate, and an inverse correlation with total MUs used. The PO algorithm offers comparable plan quality to the PRO, while minimizing MLC complexity, thereby improving the delivery efficiency and accuracy.

## 1 INTRODUCTION

In the past decade, stereotactic radiation therapy has been increasingly used in the management of both intracranial (stereotactic radiosurgery, SRS) and extracranial tumors (stereotactic body radiation therapy, SBRT). This technique is able to precisely deliver very high doses to the tumors while sparing the adjacent normal tissues in just a few fractions, and has been proven to provide superior or comparable treatment outcomes and is cost‐effective relative to alternative conventional techniques.^1^, ^2^, ^3^, ^4^, ^5^, ^6^, ^7^, ^8^, ^9^, ^10^

Several delivery options have been implemented in Linac‐based stereotactic treatments, including noncoplanar three‐dimensional conformal radiation therapy (3D‐CRT), dynamic conformal arcs (DCA), intensity modulated radiation therapy (IMRT, including sliding window and step‐and‐shoot techniques), and volumetric modulated arc therapy (VMAT). Recently DCA and VMAT have become popular due to their delivery efficiency compared to 3D‐CRT and IMRT. For DCA treatment, the multileaf collimator (MLC) is shaped to conform to the planning target volume (PTV) based on the beam's eye view (BEV) as the gantry rotates around the patient. The gantry speed and dose rate remain constant during the DCA delivery.

Volumetric modulated arc therapy was first introduced and implemented into the clinic as a novel radiation delivery technique^11^, ^12^ that was a variation on static field IMRT. Unlike DCA, VMAT plans are generated by an inverse planning algorithm, which allows modulation of the gantry rotation speed, dose rate, and also the position and speed of MLC. It is recommended that VMAT plans always be validated with a patient‐specific QA measurement before treatment. Compared to VMAT, DCA techniques offer advantages in terms of reduced multileaf collimator (MLC) motion complexity, positioning error, and delivery efficiency, thus DCA may be the desired delivery modality from a technical treatment delivery viewpoint. However, VMAT may be favored due to its increased ability for dose shaping when certain critical organs sparing becomes important.

The photon optimization (PO) algorithm was recently released by Varian Medical System (Palo Alto, CA, USA) to improve IMRT and VMAT optimizations in Eclipse treatment planning system (TPS) version 13.5. The PO algorithm combines the dose–volume optimizer (DVO) used for static field IMRT and the progressive resolution optimizer (PRO) used for VMAT plans from the previous Eclipse version. Both PRO and PO algorithms create VMAT plans based on dose–volume objectives, and generate a sequence of control points, which define MLC leaf positions and MU/degree as a function of gantry angle. For both PRO and PO algorithms, the initial conditions are defined using control points to represent each VMAT field; a multiresolution approach and an objective function (the sum of the dose–volume and user‐defined objectives) are used to optimize the plan. The multiresolution dose calculation (MRDC) algorithm is used to increase the dose calculation accuracy with progressive dose calculation segments using a point cloud‐based model.^13^ The optimization process goes through four multiresolution level, in which number of dose calculation segments increase progressively at each level. The number of control points keeps unchanged during the whole optimization process.

**Figure 1:**
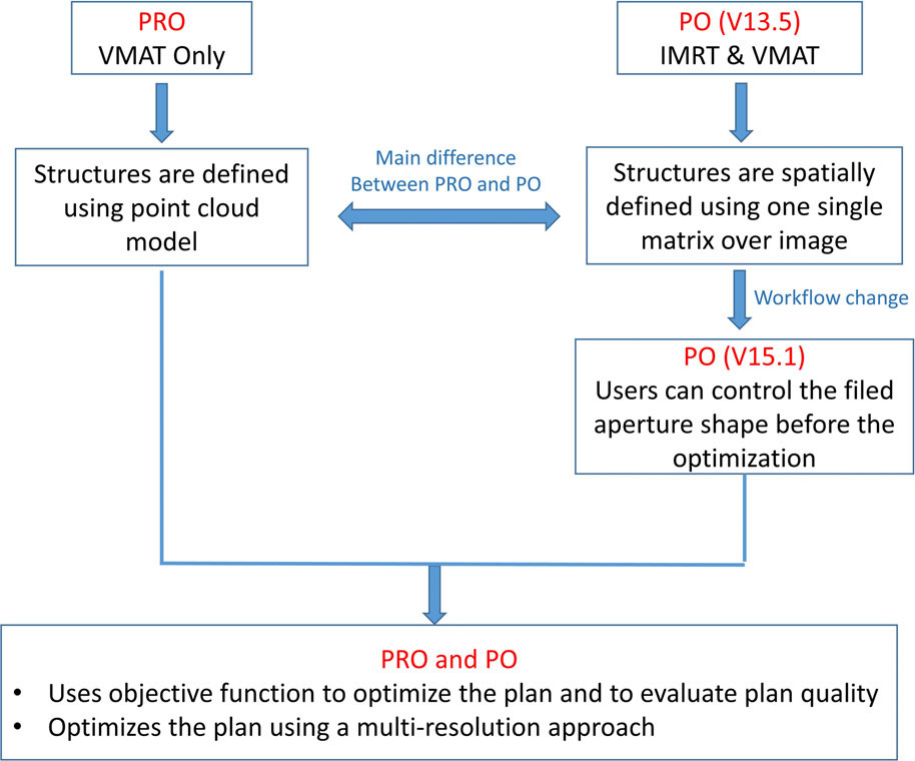
Comparisons of PRO and PO algorithms.

The main difference of the PO algorithm from the PRO algorithm is that instead of using a point cloud mode for defining structures in PRO, the PO algorithm implements a new structure model, where structures, DVH calculations, and dose sampling are defined spatially using a single matrix over the image. Fixed values (1.25 mm, 2.5 mm, or 5 mm) are used for the voxel resolution of the matrix. This resolution defines the planar X and Y resolution in the slices, and the Z resolution orthogonal to the slices is a function of chosen resolution and the slice spacing. This matrix defines the locations of the structures and the sampling of the dose, and it replaces the previously used point clouds in PRO algorithm. The DVH for the structures is evaluated using volume weights defined for each voxel. A workflow change has been made for the PO algorithm in the latest version of Eclipse TPS (version 15.1), namely, the users are allowed to control the field aperture shape (DCA in this study) before the VMAT optimization. This workflow was not supported in the previous Eclipse version for PO algorithm due to constraints of the field types being used for the optimizer. Initializing the VMAT inverse plan based on a DCA‐like formation of MLC apertures could potentially reduce the MLC modulation in the treatment planning process. Figure 1 shows the comparison between the PRO and PO algorithms. The purpose of this study is to compare the PO algorithm (version 15.1) with its predecessor, the PRO algorithm (version 13.6), in terms of MLC modulation reduction while preserving plan quality for both lung SBRT and brain SRS treatments.

## 2 METHODS

This retrospective analysis included 20 lung SBRT patients (10 received 54 Gy in 3 fractions and 10 received 50 Gy in 5 fractions) and 10 SRS patients (25 Gy in 5 fractions). All planning computer tomography (CT) images were acquired on a Philips Brilliance Big Bore CT scanner (Philips, Cleveland, OH, USA) with fields of view large enough to cover the patient and immobilization devices, the slice thickness was 2 mm for lung SBRT patients and 1 mm for SRS patients. All patients were treated on a Varian TrueBeam STx platform.

All lung SBRT patients were immobilized using a customized full‐body length vacuum bag (Bodyfix, Medical Intelligence Inc) for simulation and subsequent treatments. Paddle‐based abdominal compression was applied to restrict the tumor motion within 1.5 cm if needed. All patients underwent a free breathing and a 10‐phase four‐dimensional CT (4DCT) scan. The respiratory motion was captured with a Philips bellows system placed around the patient abdomen. An internal target volume (ITV) was created based on the maximum intensity projection (MIP) of 4DCT image, and the PTV was created by a 5 mm uniform expansion of the ITV. The original clinical plans used for treatment were created on the free‐breathing CT and involved two 6 MV flattering filter free (6X‐FFF) beams. Each clinical plan was normalized such that at least 95% of the PTV received the prescription dose, and more than 99% of the PTV received at least 90% of the prescription dose. The maximum point dose and dose–volume constraints of several critical organs are listed in Table 1 for both 54 and 50 Gy protocols.^14^, ^15^, ^16^

**Table 1:** Lung SBRT planning acceptance objectives for critical organs

Serial tissue	Volume	54 Gy		50 Gy	
		Volume max (Gy)	Max point (Gy)	Volume max (Gy)	Max point (Gy)
Spinal cord	<0.25 cc <0.5 cc		18	22.5 13.5	30
Lungs‐ITV	<15%	20		20	
Esophagus	<5 cc	17.7	25.2	19.5	35
Aorta	<10 cc	39	45	47	53
Trachea	<4 cc	15	30	16.5	40
Skin	<10 cc	30	33	36.5	39.5
Chest wall	<30 cc	30		30	

All SRS patients were immobilized in BrainLab SRS masks (BrainLab AG, Feldkirchen, Germany) and received IV contrast prior to the CT scan when possible. All patients also underwent MR scans, which were registered with the planning CT to define the gross tumor volume (GTV). The GTV was expanded by 1 mm margin uniformly to form the PTV. MR scans were acquired on a GE Signa EXCITE 3.0T MRI scanner (GE Healthcare, Waukesha, WI, USA) with 1 mm slice thickness and a field of view large enough to include the entire surface of the patient. Postcontrast T1‐weighted axial scans were used for all cases. 6X‐FFF beams with 3–4 table kicks were used for the original clinical VMAT plans, and at least 99% of PTV and 100% GTV coverages were enforced. The maximum point dose and dose–volume constraints for critical structures followed the guidelines of AAPM TG‐101.^14^

All the clinical plans were optimized in Eclipse version 13.6 with the PRO algorithm, which is based on dose–volume objectives. All plans were reoptimized with the new PO algorithm in Eclipse version 15, which offers a VMAT optimization based on an initial DCA plan as described above. The PO algorithm determines the optimal field shape and intensity by interactively conforming the dose distribution to the desired objectives until an optimum solution is reached. In the new PO algorithm, DCA was first used to conform to the target and followed by VMAT inverse planning to achieve the desired dose distribution. The DCA beams were automatically fit with 3–4 mm margin around the PTV, and the same beam configurations (gantry, collimator and table angles) were used in the re‐plan. In order to isolate the variation due to the planning optimization algorithm, the same planning objectives, constraints, and weightings for the target and critical organs were used for both the PRO and PO plans. The settings were also retained for normal tissue optimization (NTO) and the MU objectives.

PTV coverage was forced to be identical for the same patient in order to have a comparison between the PRO and PO algorithms (at least 95% for lung SBRT and 99% for SRS patients). For lung SBRT treatments, selected dose–volume parameters were compared for the PRO and PO plans, including the conformity index (*CI*_50_, volume receiving 50% of prescription dose/PTV volume) for the PTV, *V*_20_ (lung volume receives 20 Gy) for lungs, *V*_30 Gy_ for chest wall, and *D*_0.035 cc_ (dose to the 0.035 cc of the volume, a representative of maximum dose) for all other critical organs, such as spinal cord, aorta, and trachea. For brain SRS treatments, plan quality was evaluated based on the conformity index (*CI*_100_) for the PTV and normal volumes encompassed by the 12 and 6 Gy isodose lines (*V*_12_ and *V*_6_).

Treatment plan complexity was assessed by using the modulation complexity score (MCS) and average open aperture per control point (AA). The MCS was originally defined by McNiven to evaluate the plan complexity and deliverability for step‐and‐shoot IMRT plans,^17^ and then modified by Masi in order to apply for VMAT plans (considering control points of the arc instead of segments).^18^ The plan complexity analysis (MCS and AA) was implemented as a plug‐in script to the Varian Eclipse planning system. Higher MCS and AA values mean less plan complexity, and easier plan deliverability.

Agreement of the planning and delivered doses was assessed by studying the patient‐specific QA results. Patient‐specific QA was performed with ArcCHECK (Sun Nuclear) and gamma passing rates were compared between the PRO and PO plans. The criteria for gamma analysis were 3%/3 mm and 2%/2 mm for SBRT plans, and 5%/1 mm and 3%/1 mm for SRS plans with 10% threshold. The Mann–Whitney U test was used to evaluate the differences between the two groups of plans for MCS, total MUs used, gamma passing rate, and dosimetric parameters for critical structures.

All work was carried with the approval of the institutional review board under protocol number 1767.

## 3 RESULTS

The average volume of the PTV for SBRT patients was 34.1 ± 12.5 cc (range from 10.1 to 54.2 cc) and 30.8 ± 18.1 cc (range from 9.7 to 59.5 cc) for 54 and 50 Gy treatments, respectively. The average volume of the PTV was 10.1 ± 10.3 cc (range from 0.9 to 31.4 cc) for SRS patients. Table 2 lists the distribution of the tumor locations and target coverage for the group of the patients included in this analysis.

**Table 2:** Patient characteristics and PTV coverage

	Lung SBRT						Brain SRS		
	54 Gy			50 Gy			25 Gy		
Patient ID	*V*_PTV_ (cc)	Tumor Location	PTV Coverage	*V*_PTV_ (cc)	Tumor Location	PTV Coverage	*V*_PTV_ (cc)	Target Location	PTV Coverage
1	19.6	LUL	97%	19.1	LLL	97%	6.0	Pituitary	99.7%
2	54.2	LUL	96%	35.3	RUL	97%	5.2	Pituitary	99.3%
3	34.2	RML	97%	47.6	RLL	95%	31.4	Pituitary	99.0%
4	36.9	LUL	97%	59.5	RLL	97%	9.1	Orbit	99.0%
5	37.2	RUL	97%	32.5	LUL	95%	5.5	Frontal	99.5%
6	31.0	LUL	98%	9.7	LUL	95%	6.6	Parietal	99.0%
7	27.3	LUL	97%	13.2	RUL	95%	4.5	Frontal	99.5%
8	40.3	LLL	96%	54.9	RML	95%	27.0	Skull base	99.5%
9	20.2	LLL	97%	20.9	RLL	97%	4.7	Pituitary	99.5%
10	10.1	RLL	97%	15.3	LUL	97%	0.9	Pituitary	99.0%

LUL, left upper lobe; LLL, left lower lube; RUL, right upper lobe; RLL, right lower lube; RML, right middle lobe.

**Figure 2:**
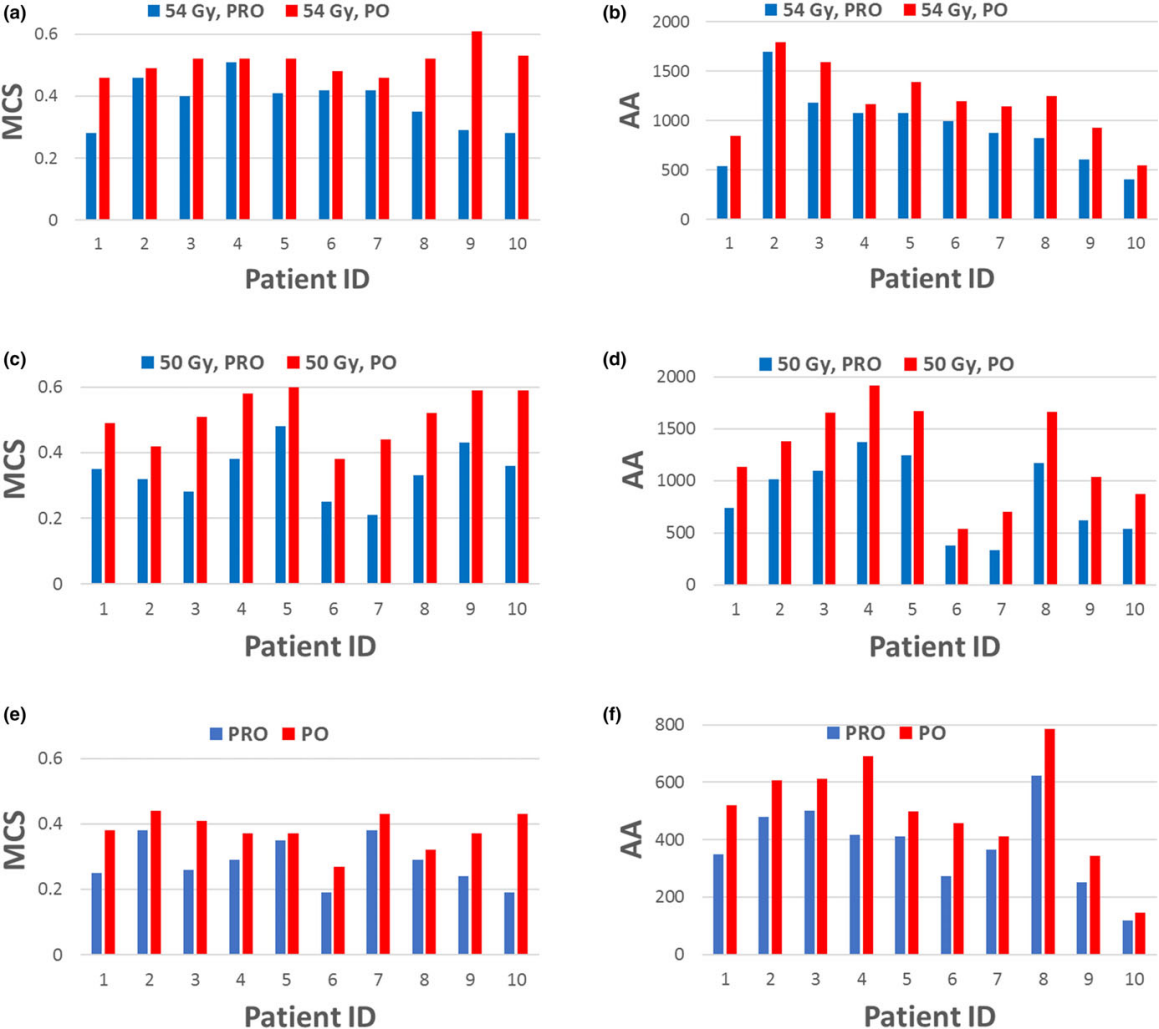
Comparisons of MCS and AA between the PRO‐ and PO plans for SBRT and SRS treatments. (a) MCS for 54 Gy SBRT; (b) AA for 54 Gy SBRT; (c) MCS for 50 Gy SBRT; (d) AA for 50 Gy SBRT; (e) MCS for SRS; (f) AA for SRS.

**Figure 3:**
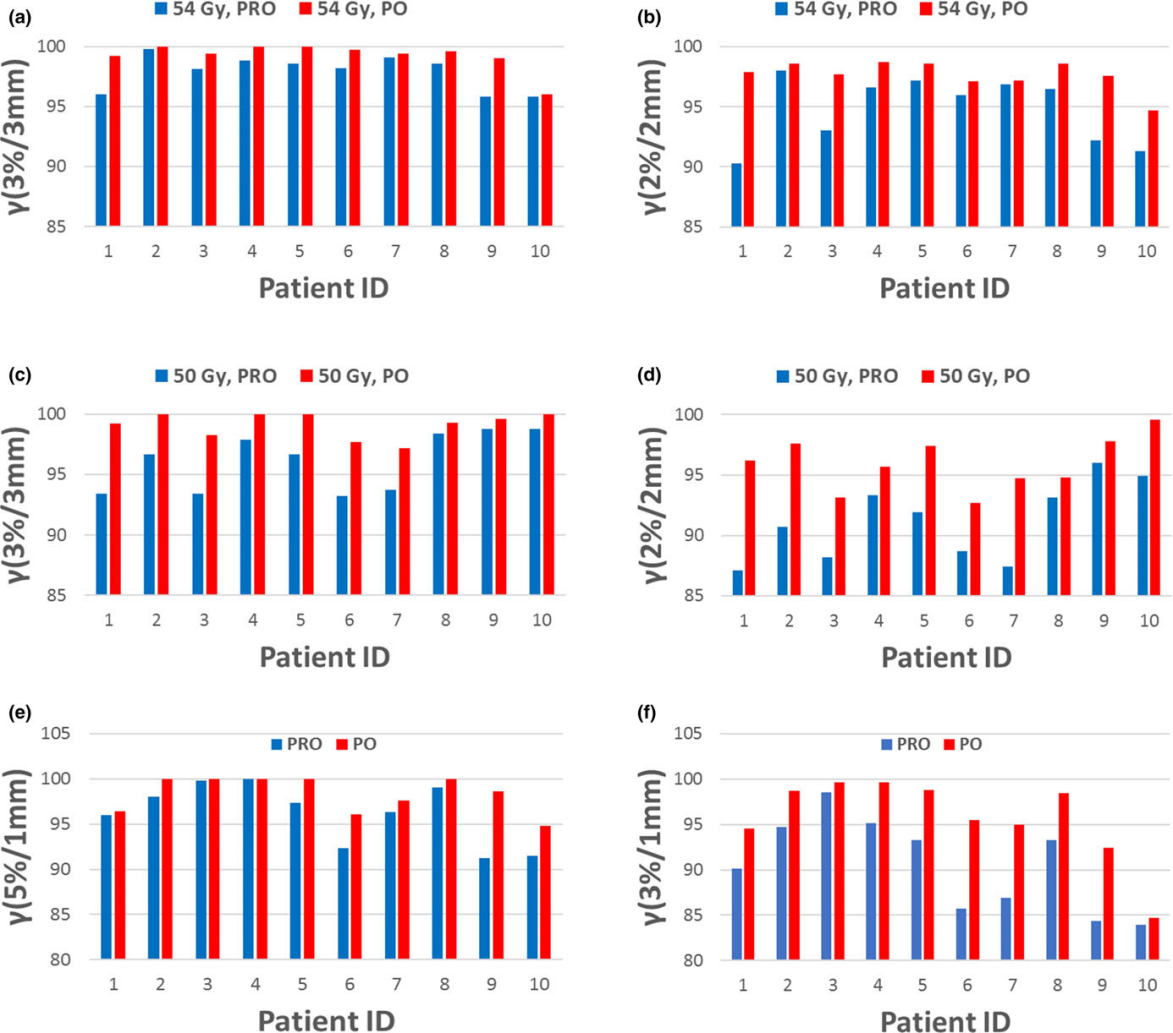
Comparisons of gamma passing rates between the PRO and PO plans for SBRT and SRS treatments. (a) 3%/3 mm for 54 Gy SBRT; (b) 2%/2 mm for 54 Gy SBRT; (c) 3%/3 mm for 50 Gy SBRT; (d) 2%/2 mm for 50 Gy SBRT; (e) 5%/1 mm for SRS; (f) 3%/1 mm for SRS.

Table 3 listed the average and standard deviations of MCS, AA, total MUs, and gamma passing rates of PRO and PO plans for both SBRT and SRS treatments. Figure 2 compares the calculated MCS and AA values for both PRO and PO plans for each individual patient. Compared to the PRO plans, MCS and AA values are larger for the PO plans, indicating PO plans have reduced plan complexity. Figure 3 compares the gamma passing rates between the PRO and PO plans for SBRT and SRS treatments with different passing criteria. Better agreement between the planned and delivered doses was found for the PO plans for all patients included in this study.

**Table 3:** Comparisons of average MCS, AA, total MUs, and gamma passing rates between the PRO and PO plans for SBRT and SRS treatments

SBRT					SRS		
Rx	54 Gy/3Fx		50 Gy/5Fx		Rx	25 Gy/5Fx	
Algorithm	PRO	PO	PRO	PO	Algorithm	PRO	PO
MCS	0.38 ± 0.08	0.51 ± 0.04	0.34 ± 0.08	0.51 ± 0.08	MCS	0.28 ± 0.07	0.38 ± 0.05
AA	927 ± 371	1184 ± 361	849 ± 375	1254 ± 469	AA	379 ± 143	507 ± 184
Total MU	5723 ± 1045	4151 ± 475	3388 ± 574	2241 ± 453	Total MU	1820 ± 600	1304 ± 473
*γ*(3%/3 mm)	97.9 ± 1.5	99.2 ± 1.2	96.1 ± 2.4	99.1 ± 1.0	*γ*(5%/1 mm)	96.1 ± 3.4	98.4 ± 2.0
*γ*(2%/2 mm)	94.8 ± 2.8	97.7 ± 1.2	91.1 ± 3.2	96.0 ± 2.2	*γ*(3%/1 mm)	90.6 ± 5.1	95.7 ± 4.6

Mann–Whitney U tests were performed between the two algorithm groups, and the resulting *P*‐values are listed in Table 4 for both SBRT and SRS treatments. No statistically significant differences between the two algorithms were found for *CI*_50_ or doses to critical structures for SBRT patients (*P >*> 0.05 from the Mann–Whitney U test). The same conclusion can be drawn with regard to the differences between two algorithms for *CI*_100_ and the ratio of *V*_12_, *V*_6_, and *V*_*3*_ for SRS patients. This indicates the PRO and PO algorithms produce very similar plan quality for the stereotactic treatments.

**Table 4:** *P*‐values from the Mann–Whitney U test for the PRO‐ and PO plans

	SBRT		SRS	
	54 Gy/3Fx	50 Gy/5Fx	25 Gy/5Fx	
MCS	0.001	0.001	MCS	0.005
Total MU	0.001	0.001	Total MU	0.024
*γ* (3%/3 mm)	0.008	0.004	*γ* (5%/1 mm)	0.097
*γ* (2%/2 mm)	0.005	0.005	*γ* (3%/1 mm)	0.026
*CI*_50_	0.596	0.289	*CI*_100_	0.364
*V*_20 Gy_ (Lung)	0.881	0.912	*V*_12_	0.968
*D*_max_ (Eso)	0.795	0.912	*V*_6_	0.728
*D*_max_ (Aorta)	0.818	0.340	*V*_3_	0.795
*D*_max_ (trach)	0.968	1.000		
*D*_max_ (Skin)	0.968	0.968		
*V*_30_ (Chest wall)	0.728	0.849		
*D*_max_ (Cord)	0.912	1.000		

As can be seen from Table 4, statistically significant improvements in the MLC modulation (larger MCS value), delivery efficiency (reduced total MUs), and better agreement between the planned and delivered doses (improved gamma passing rate) were found for the new PO method. The MCS showed a strong correlation with gamma passing rate and an inverse correlation with the total MUs used in the plan.

## 4 DISCUSSION

Currently, DCA and VMAT are two of the most frequently used techniques in stereotactic treatment planning. DCA may be considered desirable due to advantages over VMAT including minimizing the MLC motion, position error, and delivery efficiency, etc. However, in some cases, VMAT may be able to provide more conformal dose distributions and better sparing of critical organs due to its ability to modulate intensity, especially for cases where the doses to the critical structures become a concern. Varian Medical Systems has launched the PO algorithm to combine the previous DVO and PRO algorithms as of Eclipse version 13.5. The PO algorithm further improved in version 15 by allowing the user to start the VMAT optimization from DCA plans. The main difference of the PO algorithm from the earlier PRO algorithm is that PO algorithm uses a new method to model the structure, which allows the user to optimize the VMAT plans directly from DCA beams. The purpose of this study is to compare the new photon optimization algorithm using a DCA initialization with its predecessor, the progressive resolution optimizer, in terms of MLC modulation, delivery efficiency, and agreement between the planned and delivered doses for both lung SBRT and brain SRS patients.

As shown in Table 4, no statistically significant differences (*P* << 0.05 from Mann–Whitney U test) were found for any of the dosimetric parameters between the PO and PRO algorithms (the target coverages were forced to be identical for both algorithms for a similar comparison), indicating both algorithms had comparable plan quality. Compared to the PRO plans, statistical significant improvements (*P *<* *0.05) were found for the PO plans in terms of MLC modulation (MCS and AA), total MUs used, and the gamma passing rate for the patient‐specific QA.

Two metrics, MCS (between 0 and 1) and AA, were used to evaluate the overall plan complexity for both PRO and PO plans. The MCS incorporates the leaf sequence variability and the aperture area variability into the calculation. Compared to the PRO plans, an average increase in MCS values for PO plans of 0.15 (from 0.36 to 0.51) and 0.10 (from 0.28 to 0.38) were found for SBRT and SRS, and an average increase in AA values of 331 (from 888 to 1219) and 128 (from 379 to 507). This indicated PO plans have reduced plan complexity and easier plan deliverability compare to PRO plans.

PO plans have significantly smaller total MUs used than the PRO plans (total MU reduction of 29.8% for SBRT and 28.3% for SRS). Less MUs used indicating increased plan delivery efficiency, which yields reduced intrafraction motion, and also increased patient comfort. Less beam modulation of the PO plans gives less head leakage and less collimator scattering, which could have an impact on the effort to decrease out‐of‐field dose and secondary cancers.^19^

**Figure 4:**
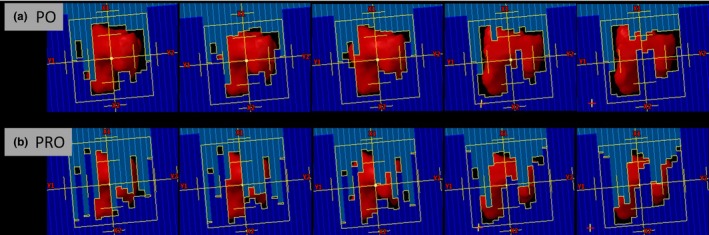
Comparisons of selective MLC openings between the PO and PRO algorithms for a representative SRS treatment. (a) PO algorithm; (b) PRO algorithm.

In our clinical practice, the guideline for passing the patient‐specific QA is *γ *> 95% at 3%/3 mm for SBRT and 5%/1 mm for SRS treatments, respectively. Better agreement between the planned and delivered doses (*γ* increased from 97.0% to 99.2% at 3%/3 mm criteria for SBRT plans and from 96.1% to 98.4% at 5%/1 mm criteria for SRS plans) were found for the PO plans. Results from tighter criteria (2%/2 mm for SBRT and 3%/1 mm for SRS) were also included in our analysis to gain more insight of the deviation of planned and delivered dose. Compared to the PRO plans, *γ* of PO plans increased from 93.0% to 96.8% at 2%/2 mm criteria for SBRT plans and from 90.6% to 95.7% at 3%/1 mm criteria for SRS plans. Better QA results from PO plans can be attributed to reduced plan modulation (MCS and AA) for the new PO algorithm, which is consistent with previous a finding.^18^ Figure 4 compares the MLC openings for five randomly selected control points between the PO and PRO plans for a representative SRS patient. We want to point out that the MLC openings for other control points have similar behaviors like those showed in the figure. One thing we noticed was the new PO algorithm reduced the small MLC opening significantly when compared to the PRO plans, this can be indicated by the increase in AA values. Small MLC opening in the VMAT plans may lead to dosimetric uncertainty due to the small field dosimetry uncertainty in the beam model. Less MUs and larger MLC openings could lead to better agreement between the planned and delivered doses in the PO algorithm, as observed by Vanetti and Giorgia.^20^, ^21^

## 5 CONCLUSION

The new PO algorithm offers comparable dosimetry to the PRO algorithm. The new PO algorithm has better gamma passing rate, indicating better agreement between the planned and delivered doses. Furthermore, the new PO algorithm has much less MLC complexity and total MUs, thereby improving the delivery efficiency and QA results. This could potentially reduce the uncertainty when considering patient interfraction motion during the course of treatment.

## CONFLICT OF INTEREST

The authors declare no conflict of interest.

## APPENDIX 1

### DEFINITION OF MODULATION METRICS (MCS) AND AVERAGE APERTURE (AA)

The MSC for VMAT plans was originally defined by McNiven for step‐and‐shoot IMRT plans,^17^ and then modified by Masi in order to apply for VMAT plans.^18^ The LSV is defined based on the difference in position for adjacent MLC leaves in the same bank for each control point (CP). The positional variations are considered relative to the maximum MLC variation per bank for each CP. The maximum variation per CP is defined as:

$$\begin{array}{c} Pos_{\max}\left(\mathrm{CP}\right)={〈\text{max}\left(pos_{n\in N}\right)-\text{min}\left(pos_{n\in N}\right)〉}_{leaf\;bank}, \end{array}$$

$$\begin{array}{cc} LSV_{\mathrm{CP}}= & {\left(\frac{\sum_{n=1}^{N-1}\left(pos_{\max}-\left|pos_{n}-pos_{n+1}\right|\right)}{\left(N-1\right)\times pos_{\max}}\right)}_{left\;bank}\\ & \times {\left(\frac{\sum_{n=1}^{N-1}\left(pos_{\max}-\left|pos_{n}-pos_{n+1}\right|\right)}{\left(N-1\right)\times pos_{\max}}\right)}_{right\;bank}, \end{array}$$

where *N* is the number of moving leaves and *pos*_*n*_ is the position of leaf *n*.

The AAV is based on the area defined by opposing MLC leaves in a CP normalized to the maximum area in the arc, which is defined by the maximum apertures for all leaf pairs over all CPs in the arc

$$\begin{array}{c} AAV_{\mathrm{CP}}=\frac{\sum_{a=1}^{A}\left({〈pos_{a}〉}_{left\;bank}-{〈pos_{a}〉}_{right\;bank}\right)}{\sum_{a=1}^{A}\left({〈\text{max}(pos_{a})〉}_{left\;bank\in arc}-{〈\text{max}(pos_{a})〉}_{right\;bank\in arc}\right)}, \end{array}$$

where *A* is the number of moving leaves in the arc.

The MLCs are continuously moving between CP while MU are being delivered, so MCS considers the mean values of *LSV*_*CP*_ and *AAV*_*CP*_ between adjacent CPs. This product is weighted by the percentage of total MU delivered between the adjacent CPs:

$$MCS=\sum_{i=1}^{I-1}\left[\frac{\left(AAV_{CP_{i}+}AAV_{CP_{i+1}}\right)}{2}\times \frac{\left(LSV_{CP_{i}+}LSV_{CP_{i+1}}\right)}{2}\times \frac{MU_{CP_{i,i+1}}}{MU_{\mathrm{arc}}}\right],$$

where I is the number of CP and MU_CPi,i+1_ are the MU delivered between two consecutive CPs.

The *AA* term is defined using the notation from above as

$$AACP=\sum_{a=1}^{A}\left({\left\langle pos_{a}\right\rangle }_{left\;bank}-{\left\langle pos_{a}\right\rangle }_{right\;bank}\right)\times w_{a}$$

where *w*_*a*_ is the width of leaf *a*

$$AA_{\mathrm{arc}}=\frac{\sum_{i=1}^{I-1}\left[\frac{\left(AACP_{CP_{i}+}AACP_{CP_{i+1}}\right)}{2}\times \frac{MU_{CP_{i,i+1}}}{MU_{\mathrm{arc}}}\right]}{I-1}.$$
